# Circumcision in Girls

**Published:** 1912-11-09

**Authors:** 


*To the Editor of* THE HOSPITAL.

### Circumcision in Girls.

*To the Editor of* THE HOSPITAL.

SIR,—As regards circumcision in girls, recommended by Dr. R. Morris, of New York, I have often thought it was a pity it was not resorted to in cases of bad masturbation, when the patient has suffered from extreme mental derangement, brought about by this habit, especially as I have seldom seen a case that has not relapsed; I also have the word of many doctors that it never is cured. When one sees how much trouble and care a patient gives to friends and attendants year after year, and the dreadful monotony of such a life, one thinks it would surely be better for an operation to be performed, and, when done, why more unpleasant than in the case of the opposite sex?—especially when it is a matter not only physical but mental. I have heard it stated, upon good authority, that it is not an unknown operation in the East. E. N.

